# Interrupted time-series analysis to evaluate the impact of a behavioral change outpatient antibiotic stewardship intervention

**DOI:** 10.1017/ash.2021.203

**Published:** 2021-11-02

**Authors:** Brittany L. Morgan, Haylee Bettencourt, Larissa May

**Affiliations:** 1 University of California Davis, School of Medicine, Public Health Sciences, Davis, California; 2 University of California Davis, School of Medicine, Department of Emergency Medicine, Sacramento, California

## Abstract

**Objective:**

We evaluated the effect of a behaviorally enhanced quality improvement intervention in reducing the number of antibiotic prescriptions written for antibiotic nonresponsive acute respiratory infections (ARIs). A secondary objective was identifying whether a reduction in inappropriate antibiotic prescriptions, if present, persisted after the immediate implementation of the intervention.

**Design:**

Nonrandomized, quasi-experimental study conducted from January 2017 through February 2020.

**Setting:**

University of California, Davis Health outpatient clinics. In total, 21 pediatric, family, and internal medicine practices in 10 cities and towns were included.

**Patients:**

Patients evaluated by a participating physician at an enrolled practice site during the study period with diagnoses (primary and secondary) from the *International Classification of Diseases, Tenth Revision* codes consistent with antibiotic nonresponsive ARI diagnoses.

**Intervention:**

A behaviorally enhanced quality improvement intervention to reduce inappropriate prescribing for antibiotic nonresponsive ARI.

**Results:**

In total, 63,028 eligible patient visits across 21 locations were included in the analysis. The most frequently prescribed antibiotic for antibiotic nonresponsive ARI was azithromycin (n = 3,551), followed by amoxicillin (n = 924). Overall, the intervention was associated with an immediate 46% reduction in antibiotic prescriptions for antibiotic nonresponsive ARI (*P* = .001) following the intervention. We detected no significant change in the month-to-month trend after the intervention was implemented (*P* = .87), indicating that the reduction was sustained throughout the postintervention period.

**Conclusion:**

Our findings demonstrate that a behaviorally enhanced quality improvement intervention to reduce inappropriate prescribing for antibiotic nonresponsive ARI in ambulatory care encounters was successful in reducing potentially inappropriate prescriptions for presumed antibiotic nonresponsive ARI.

Inappropriate use of antibiotics increases healthcare utilization and cost as well as a patient’s risk of adverse drug events and infection by *Clostridium difficile* or other multidrug-resistant pathogens, making it a major public health concern. The use of antibiotics, including inappropriate and overuse, accelerates the natural selection of antibiotic-resistant bacteria, which cause an estimated 35,000 deaths in the United States each year.^1^ Encouraging judicious prescribing of antibiotics effectively reduces unnecessary antibiotic exposure, improves antibiotic prescribing practices, and addresses the crisis of emerging antibiotic resistance.^2–5^

Antimicrobial stewardship programs are required in hospitals and long-term care facilities by the Centers for Medicare and Medicaid Services and the Joint Commission, and efforts are underway to develop programs for outpatient facilities.^6^ In 2019, ∼251 million oral antibiotic prescriptions were written in outpatient settings.^7^ The Centers for Disease Control and Prevention estimates that outpatient settings account for 85%–90% of antibiotic prescriptions in the United States, and that ∼30% of those outpatient prescriptions are unnecessary. One of the most common examples of unnecessary antibiotic prescriptions in outpatient settings is for viral respiratory infections.^8–10^ Even though ∼50% of prescriptions written for respiratory infections are inappropriate,^10,11^ programs targeting the unique needs of outpatient facilities have had limited success.

Providers in outpatient facilities face unique day-to-day challenges to antibiotic decision making. Issues such as frequent interruptions, patient expectations, and the high volumes of patients seen per hour compound with the need to make quick decisions with limited diagnostic data.^12^ Several studies have shown that interventions grounded in behavioral economics and decision science are effective in reducing inappropriate antibiotic prescribing for ARI in primary care and outpatient facilities.^13–15^ These behavioral interventions generally leverage individual accountability and social norms by utilizing feedback, nudges, and peer comparisons to improve prescribing outcomes. Peer comparisons have been shown to improve prescribing outcomes and to be sustained after the intervention for at least 12 months.^16^

In this study, we evaluated the effect of a behaviorally enhanced quality improvement intervention in reducing inappropriate antibiotic prescribing for antibiotic nonresponsive acute respiratory infections (ARIs) across a regional outpatient health system network of primary care clinics.

## Methods

### Study design and setting

This study was conducted as a nonrandomized quasi-experimental study. The intervention was implemented at University of California–Davis Health outpatient clinics and was deemed exempt from institutional review board requirements based on its designation as a quality improvement project. In total, 21 pediatric, family, and internal medicine practices in 10 cities and towns were included in the intervention roll out. The preintervention period was January–December 2017, the intervention was implemented across clinic sites from January to December 2018, and the postintervention period was January 2019 to February 2020. Data on patient visits were collected from a central electronic health record (EHR) database.

Eligible visits included those with diagnoses (primary and secondary) from the *International Classification of Diseases, Tenth Revision* (ICD-10-CM) codes consistent with antibiotic nonresponsive ARI diagnoses with consideration of secondary diagnostic codes as modifiers, adapted from the MITIGATE tool kit.^17^ The MITIGATE tool kit is based on an adaptation from evidence-based literature^15^ (see tool kit background) and the Center for Disease Control and Prevention (CDC) Core Elements of Outpatient Antibiotic Stewardship. The following conditions were targeted for reducing antibiotic prescribing by the intervention: nonsuppurative otitis media, acute nasopharyngitis, laryngitis, supraglottitis, croup, influenza, viral pneumonia, viral bronchitis, unspecified bronchitis, bronchiolitis, lower respiratory tract infection unspecified, vasomotor and allergic rhinitis, chronic nasopharyngitis, bronchitis, not specified as acute or chronic, and asthma (see MITIGATE toolkit Table 3, ICD-10 Codes List in ref. 17 for accompanying ICD-10 codes).

The parameters for identifying antibiotic nonresponsive ARIs were intended to be congruent with existing Healthcare Effectiveness Data and Information Set and National Quality Forum^18^ quality metrics on acute bronchitis. However, we broadened them to include all other antibiotic nonresponsive ARIs as well as pediatric and geriatric populations. A patient visit was eligible for inclusion if (1) the patient was evaluated by a participating physician at an enrolled practice site and was assigned an ICD-10 code consistent with antibiotic nonresponsive ARI and (2) the visit occurred during the preintervention, intervention, or postintervention period. Visits were excluded from the primary analysis if the patient had either a nonacute respiratory bacterial infection diagnosis or an antibiotic-appropriate ARI diagnosis that co-occurred with their qualifying diagnosis at the visit, such as urinary tract infections or pneumonia. The sets of exclusionary diagnoses that were used to identify antibiotic nonresponsive ARIs are listed in the public MITIGATE tool kit, with the addition of the ICD-10 for acne to include for antibiotic use concordant with guidelines for management of acne.

The primary outcome measure for this analysis was the number of all antibiotics reasonably assumed to be prescribed for an antibiotic nonresponsive ARI at each clinic, per month. Antibiotics were identified by pharmacy therapeutic class through the EHR if the prescription occurred the same day or within 3 days of the office encounter. Non–office-based visits including phone encounters, patient messaging, or orders only were excluded. If an individual was prescribed 2 antibiotics at the same encounter, both antibiotics were included in the monthly total if both were accompanied by an ICD-10 code consistent with an antibiotic-nonresponsive ARI. The study was designed to test whether the antibiotic stewardship program resulted in an immediate statistically significant decrease in the number of antibiotic prescriptions written for antibiotic-nonresponsive ARI. We also sought to determine whether a reduction in inappropriate antibiotic prescriptions, if present, persisted after the immediate implementation of the intervention.

### Antibiotic stewardship program

The adapted intervention incorporated strategies from the CDC Core Elements of Outpatient Antibiotic Stewardship, which provides a recommended framework for implementing stewardship in outpatient settings.^19^ Several of the core elements were included in the behaviorally enhanced quality improvement intervention using implementation tools feasible in the acute-care setting and accepted by local physicians. Strategies incorporated include leadership support, data reporting and feedback through clinic comparisons and physician feedback, educational strategies, and identification of a site champion.

Our program was sponsored by the leadership of the primary care clinics as well as the chief quality officer and formal outpatient antibiotic stewardship program. The intervention included educational sessions, sharing of data by clinic (comparisons), as well as patient and physician educational materials. Each month the antibiotic prescribing practices for nonresponsive ARI at each clinic were aggregated from the EHR and sent to the champions and medical directors for clinic comparisons. Champions were identified for each of the clinics by the individual site medical directors and were tasked with leading provider education and advocating for antimicrobial stewardship. Champions collaborated with clinical and operations staff to adapt each of the intervention components to ensure that they were consistent with local systems, policies, and standards. These customizations ensured that the program fit within the organization culture and workflow. A plan was developed for implementing and monitoring each of the components. Standard operating procedures were refined and shared with staff.

The intervention period began with resident education and staff meetings in January 2018. Education was provided via e-mail as well as through individual site meetings with each of the clinic providers. In one clinic, the hospital-based internal medicine clinic-public commitment posters with physician signatures were posted. Academic detailing of outlying providers using individual provider data was performed by the medical directors or champions in select clinics where baseline and subsequent antibiotic prescribing rates were higher.

### Statistical analysis

**Fig. 1.**
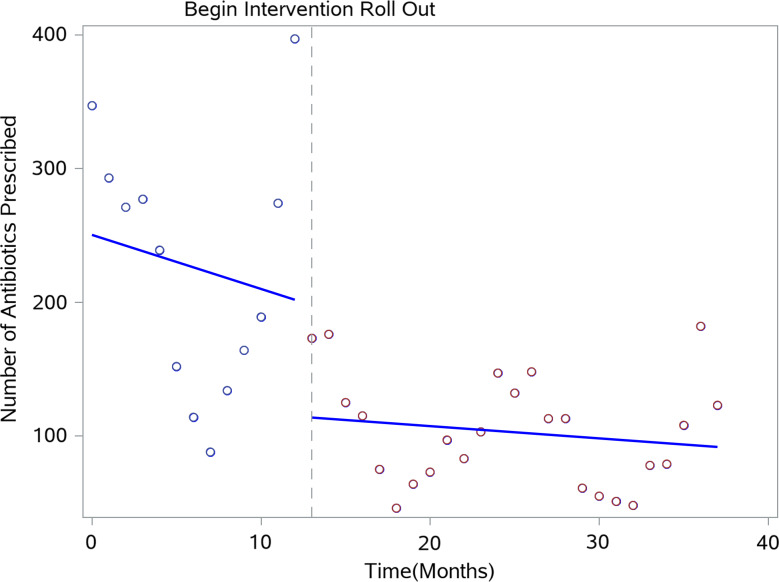
Number of total antibiotic prescriptions written for antibiotic nonresponsive acute respiratory infections during the study period across all clinics, 2017–2020.

The number of eligible visits and the outcome variable, number of antibiotic prescriptions written for antibiotic nonresponsive ARI, were aggregated at the month level, resulting in 38 data points. The rate of antibiotic nonresponsive ARI prescriptions was explored for all clinics with a scatter plot and line of best fit to visualize the slope direction and change across the study period prior to modeling (Fig. 1). Descriptive statistics including average patient age and average number of eligible monthly visits at each site were compared before and after the intervention to ensure that the assumptions for analysis were met.

The intervention effect was assessed using an interrupted time series (ITS) analysis over the study period. A sequential period variable, the outcome variable, the location-specific intervention time point, and a location-specific time after the intervention variables were included in the model. The period is a continuous variable indicating the time in months from the start of the study period. The location-specific intervention is an indicator variable for time occurring before the intervention and the time occurring after the intervention. Depending on what month the intervention was implemented for each location, each clinic has periods with intervention = 0, indicating the points in the study considered before the intervention for that specific clinic, and an intervention = 1, indicating the points in the study considered after the intervention. Time after the intervention is a continuous variable counting the number of months after the intervention implementation for each location. Seasonality was controlled for in the model using the CDC-defined peak of winter respiratory virus season, December through March.^20^

A segmented regression technique^21^ was performed using a negative binomial, mixed-effects model of the monthly data (ie, the Glimmix procedure) using SAS software (SAS Institute, Cary, NC). The intervention and time after the intervention variables accounted for the location-specific time points of implementation. Period, intervention, and time after the intervention were included as fixed effects (ie, the study-wide effects). To account for the rollout of the intervention across the clinics during the intervention period, individual clinics could have unique starting points as well as different immediate and gradual effects of the intervention by including random intercepts, intervention, and time after intervention terms. A random residual with an autoregressive variance structure was used to account for the correlation of measurements over time, across clinics, and overdispersion. The total number of eligible visits at each clinic, each month was used as an offset variable (denominator for the primary outcome of interest) in the model to compare rates.

The intervention variable was assessed to estimate the immediate intervention effect. This effect indicates the interventions’ immediate impact on antibiotic nonresponsive ARI antibiotic prescribing at a clinic and is denoted by a change in level, or y-intercept, immediately following the intervention implementation. The long-term intervention effect is assessed by evaluating the time after intervention variable. This effect indicates whether the intervention impact persisted after the immediate intervention effect into the postintervention period. The long-term intervention effect is denoted by a difference in slope from the preintervention period to the postintervention period. The differential impact of the intervention was assessed for adult and pediatric providers. Individual clinics were combined if they were in the same city or town to maintain confidentiality. All statistical tests were 2-sided and *P* values < .05 were considered significant.

## Results

### Clinic descriptions and antibiotic nonresponsive ARI antibiotic prescribing

In total, 21 clinics across 10 cities and towns were enrolled in the intervention. From January 2017 through February 2020, a total of 63,028 eligible patient visits occurred in the enrolled clinics. The average number of eligible patient visits each month was 166 visits (95% CI, 52–381) over the study period. The average patient age was 44 years (SD, 25). At the start of the study, the percentage of visits in which an antibiotic was prescribed for antibiotic nonresponsive ARI ranged from 3.7% to 34.2%. During the preintervention period, an antibiotic was prescribed, on average, for 11.5% (95% CI, 2.0–25.2) of eligible visits. By the end of the study period, the percentage of antibiotic nonresponsive ARI antibiotic prescriptions had decreased to a low of 1.2% of visits and a high of 18% of visits. During the postintervention period, an average of 5.8% (95% CI, 1.0–13.0) of eligible antibiotic nonresponsive ARI visits had an antibiotic prescribed. The most frequently prescribed antibiotic over the study period was azithromycin (n = 3,551), followed by amoxicillin (n = 924). See Table 1 for descriptive statistics and Table 2 for descriptive statistics by clinic location.

**Table 1.** Characteristics of Outpatient Prescriptions for Antibiotic Nonresponsive Acute Respiratory Infections and Patient Visits (2017–2020)

Characteristics	No.	%
**Antibiotic prescriptions**		
Amoxicillin	924	16.8
Azithromycin	3,551	64.5
Doxycycline	556	10.1
Levofloxacin	163	3.0
Sulfamethoxazole	70	1.3
Other	245	4.5
Total prescriptions	5,509	100
**Patient age**		
<18 y	15,078	24.0
≥18 y	47,950	76.0
**Encounters by location**		
A	2,760	4.4
B	3,555	5.6
C	7,369	12.1
D	7,521	11.9
E	10,515	16.7
F	2,555	4.1
G	12,522	19.9
H	4,025	6.4
I	3,334	5.3
J	8,602	13.6
Total encounters	63,028	100
**Inappropriate prescriptions (mean %, 95% CI)**		
Preintervention	11.5	2.0–25.2
Postintervention	5.8	1.0–13.0

Note. CI, confidence interval.

**Table 2.** Percent of Eligible Monthly Visits in which an Antibiotic Was Prescribed, by Location (2017–2020)

Location	Before the Intervention	After the Intervention
**A**		
Monthly visits prescribed antibiotic, mean % (SD)	6.0 (4.6)	2.9 (1.6)
Monthly visits, mean (SD)	74 (24)	70 (23)
**B**		
Monthly visits prescribed antibiotic, mean % (SD)	11.8 (4.9)	5.8 (2.6)
Monthly visits, mean (SD)	95 (25)	100 (36)
**C**		
Monthly visits prescribed antibiotic, mean % (SD)	10.6 (3.8)	6.5 (2.0)
Monthly visits, mean (SD)	204 (62)	193 (54)
**D**		
Monthly visits prescribed antibiotic, mean % (SD)	10.4 (3.3)	8.5 (2.8)
Monthly visits, mean (SD)	200 (77)	196 (62)
**E**		
Monthly visits prescribed antibiotic, mean % (SD)	14.6 (4.0)	5.8 (2.5)
Monthly visits, mean (SD)	271 (82)	279 (96)
**F**		
Monthly visits prescribed antibiotic, mean % (SD)	3.6 (2.5)	2.2 (2.4)
Monthly visits, mean (SD)	69 (18)	66 (16)
**G**		
Monthly visits prescribed antibiotic, mean % (SD)	5.4 (1.5)	3.3 (1.4)
Monthly visits, mean (SD)	327 (76)	331 (80)
**H**		
Monthly visits prescribed antibiotic, mean % (SD)	10.1 (4.6)	4.1 (2.1)
Monthly visits, mean (SD)	103 (36)	110 (30)
**I**		
Monthly visits prescribed antibiotic, mean % (SD)	20.7 (7.1)	4.7 (2.8)
Monthly visits, mean (SD)	97 (30)	83 (31)
**J**		
Monthly visits prescribed antibiotic, mean % (SD)	23.9 (7.2)	9.1 (2.8)
Monthly visits, mean (SD)	265 (76)	204 (73)

### Effect of the antibiotic stewardship program

Overall, the intervention was associated with a statistically significant decrease in antibiotic nonresponsive ARI antibiotic prescribing. The intervention impact did not differ among adult and pediatric providers (*P* = .20). The immediate intervention effect indicated a 46% reduction in antibiotic nonresponsive ARI antibiotic prescriptions or 0.54 times (95% CI, 0.42–0.66; *P* = .001) as many antibiotic nonresponsive ARI antibiotics prescribed after the intervention, after controlling for seasonality. The immediate intervention effect varied by location. Among clinics at one of the suburban locations, antibiotic prescribing immediately after the intervention was 0.56 times (95% CI, 0.39–0.81) what it was before the intervention, a 55% reduction (*P* = .001). One larger suburban location saw a statistically significant increase in the number of antibiotic nonresponsive ARI antibiotic prescriptions immediately after the intervention.

The long-term intervention effect indicated no statistically significant trend in the number of antibiotic-nonresponsive ARI antibiotics prescribed across all clinics during the postintervention period. We detected no statistically significant trend in prescribing rates during the preintervention period (slope, −0.006; 95% CI, −0.02 to 0.003; *P* = .20). The slope during the postintervention period did not statistically significantly differ from the preintervention period slope (−0.001; *P* = .87). See Table 3 for segmented regression model results.

**Table 3.** Parameter Estimates, Standard Errors, and *P* Values From Negative Binomial Segmented Regression on Monthly Antibiotic Nonresponsive Acute Respiratory Infection Prescriptions, by Location (2017–2020)

Clinic Location	Immediate Intervention Effect		Change in Slope per Month (Intervention Effect Over Time)	
	Estimate (SE)	*P* Value	Estimate (SE)	*P* Value
Overall	−0.61 (0.13)	.001	−0.01 (0.01)	.87
A	−0.02 (0.22)	.94	−0.01 (0.01)	.69
B	−0.02 (0.19)	.90	0.01 (0.01)	.57
C	0.24 (0.17)	.17	0.00 (0.01)	.77
D	0.43 (0.17)	.01	0.01 (0.01)	.41
E	−0.05 (0.17)	.77	−0.01 (0.01)	.37
F	0.08 (0.23)	.74	0.00 (0.01)	.71
G	0.14 (0.18)	.43	0.01 (0.01)	.44
H	−0.21 (0.19)	.27	0.01 (0.01)	.50
I	−0.58 (0.19)	<.001	0.01 (0.01)	.56
J	−0.01 (0.16)	.96	−0.01 (0.01)	.08

## Discussion

Antibiotic stewardship programs (ASPs) are initiatives from healthcare teams that focus on optimizing antibiotic use and are aimed at enforcing practical behavioral interventions to improve excessive antibiotic prescribing. Each ASP can be uniquely configured to target specific components throughout the prescribing process to prevent inappropriate use, ranging from simple steps (eg, patient education) to more intensive processes (eg, guideline incorporation into EHRs). Overall, there is sufficient evidence to support the effectiveness of behavioral interventions in reducing overuse of antibiotics regardless of the exact mechanism applied, though important healthcare settings have been understudied, such as the community and rural settings proposed in this protocol.^14–16,22^ In the present study, a multifaceted, behaviorally enhanced quality improvement intervention to reduce inappropriate prescribing for antibiotic nonresponsive ARI in ambulatory care encounters at UC Davis, was successful in reducing potentially inappropriate prescriptions for presumed antibiotic nonresponsive ARI. Furthermore, the effects of the intervention did not wane over time after the immediate intervention effect. We detected no statistically significant increase or rebound in antibiotic nonresponsive ARI antibiotic prescriptions during the postintervention period.

Antibiotics are lifesaving medications that are vital to the treatment of severe infections. To maintain efficacy of the antibiotic supply, emphasis has been placed on improving the judicious use of antibiotics. Unfortunately, individual patient–physician decisions are not always swayed by the knowledge that antibiotics do not treat viruses or the knowledge that overprescribing will create major public health problems down the line. Healthcare professionals have been taught to do no harm, and yet, many underestimate the potential negative effects of antibiotics and assume that these risks do not outweigh the need to treat the patient immediately. In turn, many patients feel that an antibiotic prescription is a requisite part of going to the doctor. And because physicians are increasingly judged and compensated based on patient satisfaction, they often feel pressured to prescribe antibiotics even when these medicines are not likely to work.

Our findings could be a model for other large integrated health systems in rolling out stewardship and other quality improvement interventions. Furthermore, while this intervention may not be applicable to all healthcare settings, certain key elements could be scaled for additional settings and populations. These could include the importance of leadership support as outlined in the CDC *Core Elements of Outpatient Antibiotic Stewardship*, nudging elements such as clinic comparisons with prescribing data, and antibiotic commitment posters.^22^ Also, the successful use of EHR reports in the intervention highlights the need for prioritization of information technology support to obtain data reports from EHR.

The findings of this study are promising, but the study is not without limitations. Quasi-experimental studies are strong designs when randomized controlled trials are not feasible. However, clinics in our study were not randomized, and were appointed intervention periods based on availability and convenience. A more rigorous design would have been a cluster randomized trial, but the intervention rollout was more palatable to clinical leadership. Furthermore, utilizing the rollout design rather than randomizing intervention and control clinics, supported the study goal of reducing antibiotic misuse for antibiotic nonresponsive ARIs. Additionally, we were unable to control or account for any potential Hawthorne effect, physicians changing prescribing behaviors or being influenced by hearing about the intervention prior to the implementation in their clinic. Although this could affect the results at any 1 clinic and may begin to explain the unexpected increase in antibiotic nonresponsive ARI antibiotic prescriptions following the intervention at 1 large suburban clinic, the intervention was still associated with a statistically significant decrease in antibiotic nonresponsive ARI antibiotic prescriptions. This indicates that any potential Hawthorne effect was insignificant. In addition, we did not include non–office-based visits, which may have higher rates of antibiotic prescribing, and we did not confirm viral etiology because collecting laboratory information was outside the project scope.

Further studies are needed to evaluate antibiotic prescribing for non–office-based visits and to determine the best combination of interventions to encourage judicious antibiotic prescribing in the outpatient setting. From this study, we were unable to identify what the most effective components of the intervention were. It is possible that more “social” interventions similar to the public commitment posters, peer comparisons, and champions efforts can be implemented to produce an even greater outcome regarding decreasing inappropriate antibiotic use. Individual provider feedback on the stewardship components after the intervention could strengthen our understanding of how to best develop and implement behavioral interventions. Stewardship programs should work toward decreasing antibiotic overuse while maintaining a good patient–physician relationship. Patients often demand medication despite the inappropriateness of the prescription, so efforts should focus on facilitating better communication between the prescriber and the patient. Finally, reducing inappropriate antibiotic prescribing for nonresponsive ARI was the primary goal of this study, and we did not focus on the selection of particular agents. However, azithromycin was the most prescribed antibiotic over the study period, and other studies have reported that >50% of macrolide prescriptions may be inappropriate.^23^ Agent-specific interventions could be beneficial in reducing inappropriate antibiotic prescribing. The impact of agent-based stewardship programs should be assessed and compared with stewardship programs focused solely on the decision to treat or not to treat.

## References

[1] Antibiotic resistance threats in the United States, 2019. Centers for Disease Control and Prevention website. https://www.cdc.gov/drugresistance/pdf/threats-report/2019-ar-threats-report-508.pdf doi:10.15620/cdc:82532. Published 2019. Accessed October 4, 2021.

[2] Lai C-C , Shi Z-Y , Chen Y-H , Wang F-D. Effects of various antimicrobial stewardship programs on antimicrobial usage and resistance among common gram-negative bacilli causing health care-associated infections: a multicenter comparison. J Microbiol Immunol Infect 2016;49:74–82. 26586483 10.1016/j.jmii.2015.05.011

[3] Buising KL , Thursky KA , Robertson MB , et al. Electronic antibiotic stewardship—reduced consumption of broad-spectrum antibiotics using a computerized antimicrobial approval system in a hospital setting. J Antimicrob Chemother 2008;62:608–616. 18550680 10.1093/jac/dkn218

[4] Timbrook TT , Hurst JM , Bosso JA. Impact of an antimicrobial stewardship program on antimicrobial utilization, bacterial susceptibilities, and financial expenditures at an academic medical center. Hosp Pharm 2016;51:703–711. 27803499 10.1310/hpj5109-703 PMC5080988

[5] Bantar C , Sartori B , Vesco E , et al. A hospitalwide intervention program to optimize the quality of antibiotic use: impact on prescribing practice, antibiotic consumption, cost savings, and bacterial resistance. Clin Infect Dis 2003;37:180–186. 12856209 10.1086/375818

[6] New antimicrobial stewardship standard. The Joint Commission website. https://www.jointcommission.org/-/media/enterprise/tjc/imported-resource-assets/documents/new_antimicrobial_stewardship_standardpdf.pdf?db=web&hash=69307456CCE435B134854392C7FA7D76. Accessed January 14, 2021.

[7] Outpatient antibiotic prescriptions—United States, 2019. Centers for Disease Control and Prevention website. https://www.cdc.gov/antibiotic-use/data/report-2019.html. Published 2019. Accessed October 4, 2021.

[8] Havers FP , Hicks LA , Chung JR , et al. Outpatient antibiotic prescribing for acute respiratory infections during influenza seasons. JAMA Netw Open 2018;1(2):e180243. 30646067 10.1001/jamanetworkopen.2018.0243 PMC6324415

[9] Shaver AL , Jacobs DM , LaMonte MJ , Noyes K. Antibiotic prescribing for acute respiratory tract infections in the United States outpatient setting. BMC Fam Pract 2019;20:91. 31266449 10.1186/s12875-019-0980-1 PMC6607511

[10] Fleming-Dutra KE , Hersh AL , Shapiro DJ , et al. Prevalence of inappropriate antibiotic prescriptions among US ambulatory care visits, 2010–2011. JAMA 2016;315:1864–1873. 27139059 10.1001/jama.2016.4151

[11] FastStats: emergency department visits. National Center for Health Statistics. Centers for Disease Control and Prevention website. https://www.cdc.gov/nchs/fastats/emergency-department.htm. Published November 10, 2020. Accessed January 14, 2021.

[12] Jeffs L , McIsaac W , Zahradnik M , et al. Barriers and facilitators to the uptake of an antimicrobial stewardship program in primary care: a qualitative study. PLoS One 2020;15(3):e0223822. 32134929 10.1371/journal.pone.0223822 PMC7059986

[13] Persell SD , Doctor JN , Friedberg MW , et al. Behavioral interventions to reduce inappropriate antibiotic prescribing: a randomized pilot trial. BMC Infect Dis 2016;16:373. 27495917 10.1186/s12879-016-1715-8 PMC4975897

[14] Yadav K , Meeker D , Mistry RD , et al. A multifaceted intervention improves prescribing for acute respiratory infection for adults and children in emergency department and urgent care settings. Acad Emerg Med 2019;26:719–731. 31215721 10.1111/acem.13690 PMC8146207

[15] Meeker D , Linder JA , Fox CR , et al. Effect of behavioral interventions on inappropriate antibiotic prescribing among primary care practices: a randomized clinical trial. JAMA 2016;315:562. 26864410 10.1001/jama.2016.0275 PMC6689234

[16] Linder JA , Meeker D , Fox CR , et al. Effects of behavioral interventions on inappropriate antibiotic prescribing in primary care 12 months after stopping interventions. JAMA 2017;318:1391. 29049577 10.1001/jama.2017.11152 PMC5818848

[17] May L , Yadav K , Gaona SD , et al. MITIGATE antimicrobial stewardship toolkit. SHEA website. http://shea-online.org/images/priority-topics/MITIGATE_TOOLKIT_final.pdf. Published 2018. Accessed October 4, 2021.

[18] HEDIS and performance measurement. National Committee for Quality Assurance website. https://www.ncqa.org/hedis/. Accessed May 3, 2021.

[19] Sanchez GV , Fleming-Dutra KE , Roberts RM , Hicks LA. Core elements of outpatient antibiotic stewardship. MMWR Recomm Rep 2016;65(6):1–12. 10.15585/mmwr.rr6506a1 27832047

[20] The flu season. Centers for Disease Control and Prevention website. https://www.cdc.gov/flu/about/season/flu-season.htm. Published September 3, 2020. Accessed January 14, 2021.

[21] Wong EC , Chen P-H , Hung D. Analyzing phased interventions with segmented regression and stepped wedge designs. Lex Jansen website. https://www.lexjansen.com/wuss/2014/74_Final_Paper_PDF.pdf. Published 2014. Accessed October 4, 2021.

[22] Meeker D , Knight TK , Friedberg MW , et al. Nudging guideline-concordant antibiotic prescribing: a randomized clinical trial. JAMA Intern Med 2014;174:425–431. 24474434 10.1001/jamainternmed.2013.14191 PMC4648560

[23] Sanchez GV , Shapiro DJ , Hersh AL , Hicks LA , Fleming-Dutra KE. Outpatient macrolide antibiotic prescribing in the United States, 2008–2011. Open Forum Infect Dis 2017;4(4):ofx220. 29255725 10.1093/ofid/ofx220 PMC5729700

